# Meta-analysis of structural and functional brain abnormalities in schizophrenia with persistent negative symptoms using activation likelihood estimation

**DOI:** 10.3389/fpsyt.2022.957685

**Published:** 2022-09-27

**Authors:** Tingting Zhu, Zixu Wang, Chao Zhou, Xinyu Fang, Chengbing Huang, Chunming Xie, Honglin Ge, Zheng Yan, Xiangrong Zhang, Jiu Chen

**Affiliations:** ^1^Department of Geriatric Psychiatry, The Affiliated Brain Hospital of Nanjing Medical University, Nanjing, China; ^2^Department of Psychiatry, The Third People’s Hospital of Huai’an, Huaian, China; ^3^Department of Neurology, Affiliated ZhongDa Hospital, School of Medicine Southeast University, Nanjing, China; ^4^Institute of Neuropsychiatry, The Affiliated Brain Hospital of Nanjing Medical University, Nanjing, China; ^5^The Affiliated Xuzhou Oriental Hospital of Xuzhou Medical University, Xuzhou, China

**Keywords:** persistent negative symptoms, schizophrenia, structural MRI, functional MRI, meta-analysis

## Abstract

**Background:**

Persistent negative symptoms (PNS) include both primary and secondary negative symptoms that persist after adequate treatment, and represent an unmet therapeutic need. Published magnetic resonance imaging (MRI) evidence of structural and resting-state functional brain abnormalities in schizophrenia with PNS has been inconsistent. Thus, the purpose of this meta-analysis is to identify abnormalities in structural and functional brain regions in patients with PNS compared to healthy controls.

**Methods:**

We systematically searched PubMed, Web of Science, and Embase for structural and functional imaging studies based on five research methods, including voxel-based morphometry (VBM), diffusion tensor imaging (DTI), functional connectivity (FC), the amplitude of low-frequency fluctuation or fractional amplitude of low-frequency fluctuation (ALFF/fALFF), and regional homogeneity (ReHo). Afterward, we conducted a coordinate-based meta-analysis by using the activation likelihood estimation algorithm.

**Results:**

Twenty-five structural MRI studies and thirty-two functional MRI studies were included in the meta-analyses. Our analysis revealed the presence of structural alterations in patients with PNS in some brain regions including the bilateral insula, medial frontal gyrus, anterior cingulate gyrus, left amygdala, superior temporal gyrus, inferior frontal gyrus, cingulate gyrus and middle temporal gyrus, as well as functional differences in some brain regions including the bilateral precuneus, thalamus, left lentiform nucleus, posterior cingulate gyrus, medial frontal gyrus, and superior frontal gyrus.

**Conclusion:**

Our study suggests that structural brain abnormalities are consistently located in the prefrontal, temporal, limbic and subcortical regions, and functional alterations are concentrated in the thalamo-cortical circuits and the default mode network (DMN). This study provides new insights for targeted treatment and intervention to delay further progression of negative symptoms.

**Systematic review registration:**

[https://www.crd.york.ac.uk/prospero/], identifier [CRD42022338669].

## Introduction

Schizophrenia is a severe mental illness characterized by positive and negative symptoms. Negative symptoms, including blunted affect, alogia, asociality, anhedonia, and avolition (1), have often been found to contribute to poor community and social functioning and negatively influence recovery and general health outcomes (2). Due to the significance of negative symptoms in schizophrenia, Buchanan (3) coined the term persistent negative symptoms (PNS) to describe negative symptoms that are enduring, trait-like and resistant to currently available treatments. Previous studies have indicated that the estimated prevalence of PNS is above 20% amongst patients with schizophrenia, and 23–40% in first episode psychosis (4, 5). Therefore, it is crucial to develop effective diagnosis and appropriate interventions for schizophrenia patients with PNS, which could remediate the substantial functional disability exhibited by these patients.

PNS represents a broader concept that requires at least moderate negative symptoms, a defined threshold of positive symptoms, none or low depressive and extrapyramidal symptoms (all defined on validated scales), with demonstrated clinical stability (3). The National Institute of Mental Health consensus statement recommends the use of PNS criteria in clinical research designs, especially those targeting therapeutic interventions (1). However, there is currently no assessment instrument specifically designed for PNS. Commonly accepted and validated rating scales, such as the Scale for the Assessment of Negative Symptoms (SANS) (6), Positive and Negative Syndrome Scale (PANSS) (7), Negative Symptoms Assessment (8) or newer scales like the Brief Negative Symptom Scale (9) and Clinical Assessment Interview for Negative Symptoms (10) are often used instead. Different researchers have employed distinct scales with diverse criteria to identify PNS, leading to heterogeneous results.

Magnetic resonance imaging (MRI) research has offered a significantly advanced understanding of brain structural and functional changes associated with schizophrenia (11). In recent years, advances in clinical brain imaging research have been made possible by improvements in the measurement of the distinct aspects of brain anatomy and function. However, the generalization of Task-based findings is limited since different groups utilized various tasks to capture a wide range of emotional states and behaviors. Therefore, for this present review, we chose to focus on task-free studies—namely structure (volume and morphometry), structural connectivity, and resting-state functional MRI findings, which are stable across mental states and hence allow better comparability across independent study settings and populations.

Structural MRI analytic approaches which are used to quantify brain abnormalities include voxel-based morphometry (VBM) for gray matter volume (GMV) and diffusion tensor imaging (DTI) for white matter. The VBM technique involves spatial normalization of the MRI structural images, extraction of gray matter from the normalized images, smoothing, and finally, statistical analyses comparing healthy controls (HCs) and patients (12). Numerous studies have indicated structural alterations in the prefrontal lobes, temporal lobes and limbic regions in schizophrenia (13, 14) which were associated with the severity of negative symptoms (15–17). DTI is also a non-invasive brain imaging method that allows indirect measurements of white matter microstructure by recording the diffusion of water molecules (18). Fractional anisotropy (FA) is the most commonly used index that quantifies the directionality of water diffusion in fiber bundles (19). A recent study reported that the FA value between the right caudate nucleus and putamen was inversely correlated with negative symptoms in schizophrenia (20), while a prior experiment found that the FA value of the anterior part of the corpus callosum was negatively correlated with the avolition score in schizophrenia (21). The inconsistency of these results demonstrates the need to evaluate structural changes in schizophrenia patients with PNS.

Resting-state functional MRI analytical methods that define the local features of the spontaneous blood oxygen level-dependent signal include the amplitude of low-frequency fluctuation (ALFF)/fractional amplitude of low-frequency fluctuation (fALFF) and regional homogeneity (ReHo). ALFF quantifies the intensity of low-frequency oscillations in spontaneous neural activity, which pinpoints the spontaneous neural activity of specific regions and physiological states of the brain (22). fALFF is defined as the total power in the low-frequency range (0.01–0.1 Hz) relative to the total power across all measurable frequencies. As such, fALFF is a normalized version of ALFF and is less susceptible to artifactual signals in regions located within the vicinity of vessels and/or significant pulsatile motion (23). Although many previous studies have found ALFF alternations in schizophrenia, including increased or decreased ALFF in the cingulate gyrus, temporal gyrus, lentiform nuclei, inferior parietal lobes and frontal gyrus (24–27), few studies have been carried out in schizophrenia patients with PNS. ReHo assumes that a given voxel is temporally similar to those of its neighbors, and can be used to detect the localized functional connectivity or synchronization of information processing with little interference from external stimuli (28). Moreover, increasing evidence shows that local functional homogeneity has neurobiological relevance to anatomical, developmental and neurocognitive factors, which could serve as a neuroimaging marker to investigate the human brain function, behaviors and neuropsychiatric disorder (29, 30). In fact, ReHo analysis has been successfully used to detect the abnormalities of regional functional synchronization in subjects with different psychiatric disorders (31–33). A recent ReHo study demonstrated that hyperactivation in the right inferior frontal gyrus/insula was positively associated with negative symptom scores (34). Resting-state functional connectivity (FC) is a powerful and reliable analysis method in which synchronous activity of brain regions can be examined in task-free conditions (35). FC is particularly useful in elucidating patterns of functional integration throughout the brain (i.e., how different brain regions function together) (36). Resting-state studies in schizophrenia have reported increased FC in the left orbital medial frontal cortex and right putamen regions, and reduced FC between the striatum and the right medial orbitofrontal cortex, which were significantly associated with negative symptom severity (37, 38). These findings from functional MRI studies using ALFF, ReHo, or FC support the statement that negative symptoms are associated with aberrant activation or dysconnectivity in extensive brain regions.

Published meta-analyses of VBM studies have focused more on alternations of GMV in schizophrenia patients (39, 40) or the relationship between GMV changes and positive symptoms, such as hallucinations (41, 42). Similarly, numerous meta-analyses have shown an activation or inactivation of functional connectivity in different brain regions in schizophrenia (43–47). However, the meta-analysis of structural and functional MRI studies in patients with PNS is limited. Only one meta-analysis of VMB studies focused on schizophrenia with PNS, and it reveals reduced GMV in the brain regions of the reward network, especially the left caudate nucleus (48). While a large number of existing negative symptom imaging studies have analyzed the relationship between structural or functional brain abnormalities and negative symptoms in schizophrenia patients from a symptomatological perspective, there is a paucity of studies pertaining to the differences in structural and functional brain alterations between the PNS subgroup and HCs. Therefore, this review aims to examine brain regions that show alterations in either structure or function in schizophrenia with PNS *via* a meta-analysis of structural MRI and functional MRI studies.

## Materials and methods

### Data sources and searches

**FIGURE 1:**
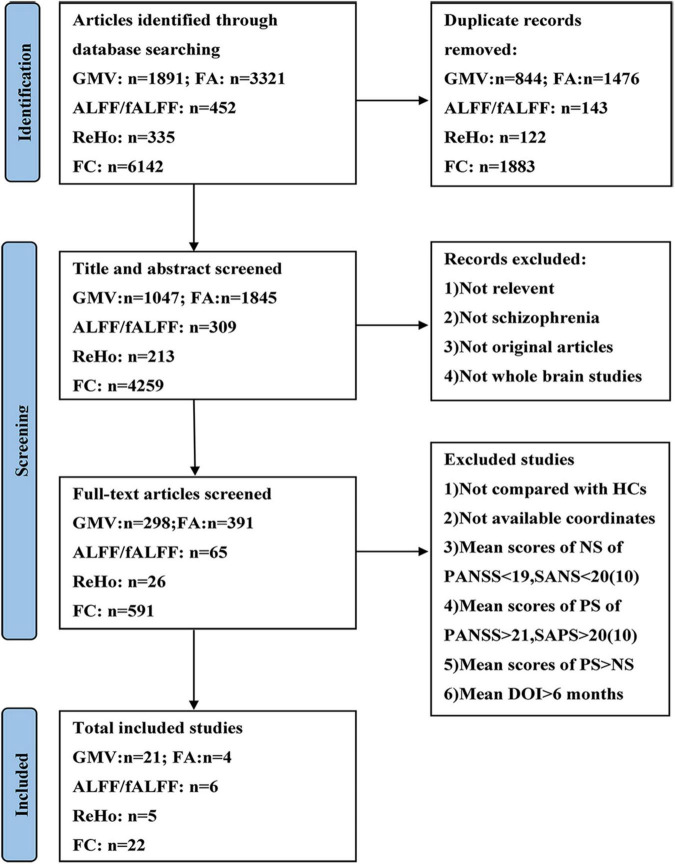
Flow diagram showing the process of identifying relevant studies. GMV, gray matter volume; FA, fractional anisotropy; ALFF, amplitude of low-frequency fluctuation; fALFF, fractional amplitude of low-frequency fluctuation; ReHo, regional homogeneity; FC, functional connectivity; HCs, healthy controls; NS, negative symptoms; PS, positive symptoms; PANSS, Positive and Negative Syndrome Scale; SANS, Scale for the Assessment of Negative Symptoms; SAPS, Scale for the Assessment of Positive Symptoms; DOI, duration of illness.

The current meta-analysis was performed according to the Preferred Reporting Items for Systematic reviews and Meta-Analyses guidelines (PRISMA) (49). A systematic selection of appropriate peer-reviewed studies was undertaken by searching the databases of PubMed, Web of Science, and Embase databases for structural and functional imaging studies. Search keywords were as follows: (1) [(gray matter) OR (cerebellar gray matter)] AND [(schizophrenia) AND (voxel-based morphometry)]; (2) [(white matter) AND (schizophrenia)] AND (diffusion tensor imaging); (3) [(functional magnetic resonance imaging) OR (RESTING STATE)] AND [(schizophrenia) AND (functional connectivity)]; (4) [(functional magnetic resonance imaging) OR (RESTING STATE)] AND [(schizophrenia) AND (regional homogeneity)]; (5) [(functional magnetic resonance imaging) OR (RESTING STATE)] AND [(schizophrenia) AND ((fractional amplitude of low frequency fluctuation) OR (amplitude of low frequency fluctuation))]. We included studies published in these databases up to September 2021. Figure 1 shows the flowchart of the literature search and eligibility assessment.

### Eligible criteria and quality assessment

Inclusion and exclusion criteria were adopted to screen literature. The following inclusion criteria were used to select eligible studies: (1) original articles written in English; (2) the schizophrenia diagnosis for patients was based on DSM criteria (negative symptoms must be measured by validated rating scales such as the PANSS and SANS, and must reach at least mild or moderate severity); (3) the duration of illness must be longer than or equal to 6 months; (4) used whole-brain structural imaging (VBM and DTI) or functional imaging (ALFF/fALFF, ReHo, FC) in schizophrenia patients; (5) reported whole-brain results in stereotactic (*x, y, z*) coordinates; (6) compared schizophrenia subjects with HCs; and (7) aged 19 years and above.

Before Buchanan developed the criteria of PNS, many researchers used different terms and criteria to identify patients with PNS, which complicated the search. To address this problem, we adopted the following exclusion criteria formulated by Li et al. (48) to identify the relevant studies: (1) mean PANSS negative score of <19, or mean SANS total score of <20; (2) mean PANSS positive subscale score of >21, or the mean Assessment of Positive Symptoms (SAPS) total score of >20; (3) studies in which the mean positive symptom scores exceeded the mean negative symptom scores.

Two authors (Tingting Zhu and Zixu Wang) independently selected eligible studies according to the abovementioned criteria and assessed the quality of the included studies. A 12-point checklist was used to estimate the quality of each included study, based on the reported demographic and clinical characteristics of the participants and the imaging methodology (50). Each point was scored as 0, 0.5, or 1 if the criteria were unfulfilled, partially met or fully met, respectively, and any study scoring >6.0 was included in the meta-analysis (see Supplementary material).

### Data extraction

The research results were screened independently by two authors (Tingting Zhu and Zixu Wang) according to the inclusion and exclusion criteria. In case of disagreement, the reviewers (Xiangrong Zhang and Jiu Chen) evaluated and made the final decision. Preliminary screening of the titles and abstracts was conducted so as to evaluate whether they conform to the research content being explored. For articles that conformed to the research content or with content that could not be determined according to the title and abstract, the full text was reviewed for a more extensive assessment. Articles obtained after the preliminary screening were re-examined to assess whether they meet the inclusion criteria. Finally, we crosschecked the references of all the retrieved results to find any missing studies.

### Data analysis

Ginger ALE version 2.3.6^1^ was used for the coordinate-based meta-analyses of the neuroimaging data. The algorithm estimated the convergence of activation based on significant foci extracted from selected studies. Localization probability distributions for all foci were modeled as the center of 3D Gaussian functions. The width of the Gaussian probability distribution was determined individually for each experiment based on empirical estimates of between-subject variability taking into account the number of subjects in each experiment (51). Gaussian distributions were pooled voxel-wise within experimental contrasts and across contrasts within a group to create a whole-brain ALE map. Within this whole-brain ALE map, each voxel was assigned a unique ALE value that represents the likelihood of experimental effects in that voxel (52). For ALE Map creation, coordinates and cluster sizes associated with significant activation or deactivation were first converted to Talairach space using the MNI to Talairach conversion tool provided by the Ginger ALE toolbox. The false discovery rate method was employed to correct for multiple comparisons at a significance threshold (*p* < 0.01, 1000 permutations). ALE results were overlaid into the MNI 152 template and viewed using the Mango^2^ and DPABI software^3^.

To test the replicability of the results, we performed a systematic whole-brain jackknife sensitivity analysis in the meta-analysis by repeating the main analysis *n* times (*n* = the number of datasets included), dropping one study at a time to determine whether the results remained detectable. However, due to the limitations in the number of included studies involving different metrics, sensitivity analyses were performed only for the group with GMV and FC in the PNS patients.

## Results

### Search results

The search results and inclusion procedures are shown in Figure 1. The study characteristics and results are summarized in Tables 1, 2. A total of 57 studies were eventually eligible for inclusion and quality assessment. Among these studies, 21 used VBM to analyze gray matter abnormalities and four studies employed DTI to examine white matter abnormalities and the remaining 32 articles comprised resting-state functional MRI studies (6 used the ALFF method, 5 employed the ReHo method, and 22 utilized whole-brain FC method). The results of the quality assessment and jackknife sensitivity analysis are available in the Supplementary material.

**TABLE 1:** Demographic and clinical information for the structural MRI studies included in the meta-analysis.

Study	Subjects (*n*)	Gender (F/M)	Age (mean ± SD)	NS scale	PS/NS	Duration	Scanner	Thickness	Coordinates	Foci.no
**GMV**										
Paillère-Martinot et al. (117)	SZ (20) HC (20)	SZ (0/20) HC (0/20)	SZ (29 ± 7.2) HC (26 ± 6)	PANSS	PS (17.3) NS (27.6)	10 years	1.5	1.5	Talairach	9
Sigmundsson et al. (118)	SZ (27) HC (27)	SZ (1/26) HC (2/25)	SZ (34.9 ± 7.6) HC (32.2 ± 6.7)	PANSS	PS (14.7) NS (25)	13.9 years	1.5	3.0	Talairach	4
Kawasaki et al. (119)	SZ (25) HC (50)	SZ (11/14) HC (22/28)	SZ (25.8 ± 4.5) HC (24 ± 5.7)	SANS	SAPS (5.6) SANS (10.3)	3.1 years	1.5	1.0	Talairach	19
Jayakumar et al. (120)	SZ (18) HC (18)	SZ (9/9) HC (9/9)	SZ (24.9 ± 6.3) HC (25.7 ± 7.5)	PANSS	PS (19) NS (23)	10.3 months	1.5	1.0	Talairach	10
Bassitt et al. (121)	SZ (50) HC (30)	SZ (12/38) HC (9/21)	SZ (31.7 ± 7.1) HC (31.2 ± 7.6)	PANSS	PS (12.9) NS (19.8)	11.4 years	1.5	NA	Talairach	4
Koutsouleris et al. (15)	SZ (59) HC (177)	SZ (9/50) HC (54/123)	SZ (32.8 ± 10.3) HC (31.5 ± 9.2)	PANSS	PS (13.0) NS (26.6)	1.9 years	1.5	1.5	MNI	26
Meisenzahl et al. (122)	SZ (72) HC (177)	SZ (16/56) HC (54/123)	SZ (35.6 ± 10.3) HC (31.5 ± 9.2)	PANSS	PS (17.9) NS (25.1)	114.4 months	1.5	1.5	MNI	67
Herold et al. (123)	SZ (18) HC (21)	SZ (7/11) HC (10/11)	SZ (28.7 ± 10.3) HC (27.4 ± 6.5)	PANSS	PS (14.2) NS (19.6)	3.4 years	1.0	2.0	MNI	38
Whitford et al. (124)	SZ (31) HC (21)	SZ (11/20) HC (9/12)	SZ (19.3 ± 3.5) HC (19.6 ± 4.3)	PANSS	PS (18) NS (20)	6.4 months	1.5	NA	Talairach	1
Cascella et al. (125)	SZ (19) HC (90)	SZ (3/16) HC (47/43)	SZ (35.1 ± 11.9) HC (46.3 ± 12.7)	SANS	SAPS (3.4) SANS (17.0)	11.8 years	1.5	1.5	Talairach	14
Anderson et al. (126)	SZ (15) HC (20)	SZ (2/13) HC (3/17)	SZ (34.3 ± 7.1) HC (33.3 ± 8.4)	PANSS	PS (13.0) NS (20.0)	11.4 years	3.0	NA	MNI	7
Huang et al. (127)	SZ (18) HC (18)	SZ (9/9) HC (9/9)	SZ (22.67 ± 3.85) HC (25.06 ± 2.44)	PANSS	PS (18.61) NS (22.06)	12.44 months	3.0	1.0	MNI	5
Poletti et al. (128)	SZ (96) HC (136)	SZ (29/67) HC (68/68)	SZ (37.2 ± 9.33) HC (33.3 ± 12.97)	PANSS	PS (17.62) NS (20.41)	12.61 years	3.0	0.8	MNI	8
Huang et al. (129)	SZ (24) HC (26)	SZ (10/14) HC (9/17)	SZ (24.25 ± 6.64) HC (23.15 ± 5.36)	SANS	SAPS (19.38) SANS (26.79)	9.28 months	3.0	1.0	MNI	3
Kim et al. (56)	SZ (22) HC (22)	SZ (10/12) HC (10/12)	SZ (31.7 ± 10.1) HC (31.6 ± 9.5)	PANSS	PS (18.4) NS (21.1)	9.2 years	3.0	NA	MNI	4
Kuroki et al. (130)	SZ (15) HC (23)	SZ (0/15) HC (0/23)	SZ (44.1 ± 9.2) HC (37.9 ± 9.1)	PANSS	PS (14.3) NS (19.7)	18.0 years	3.0	0.6	MNI	9
Szendi et al. (66)	SZ (8) HC (13)	SZ (2/6) HC (7/6)	SZ (34) HC (34)	PANSS	PS (17.5) NS (27.5)	13 years	1.5	1.0	MNI	9
Spalthoff et al. (131)	SZ (51) HC (102)	SZ (17/34) HC (33/69)	SZ (35.2 ± 10.9) HC (33.2 ± 9.6)	SANS	SAPS (19.38) SANS (42.45)	8.8 years	3.0	NA	MNI	6
Zhao et al. (132)	SZ (41) HC (39)	SZ (19/22) HC (20/19)	SZ (34.8 ± 7.1) HC (22.2 ± 4.6)	PANSS	PS (19.9) NS (20.2)	14.2 years	3.0	1.2	MNI	8
Lei et al. (133)	SZ (14) HC (32)	SZ (4/10) HC (9/23)	SZ (21.8 ± 5.35) HC (21.6 ± 4.65)	PANSS	PS (20.93) NS (26.86)	1.11 years	3.0	1.0	MNI	1
Neugebauer et al. (134)	SZ (18) HC (19)	SZ (7/11) HC (7/12)	SZ (36.94 ± 9.9) HC (35.8 ± 11.56)	PANSS	PS (15.33) NS (23.61)	12.58 years	3.0	1.0	MNI	21
**FA**										
Rametti et al. (135)	SZ (25) HC (24)	SZ (13/12) HC (13/11)	SZ (32.2 ± 6.8) HC (31.8 ± 7.0)	PANSS	PS (16.88) NS (22.52)	10.42 years	1.5	1.5	MNI	1
Spalletta et al. (136)	SZ (21) HC (21)	SZ (2/19) HC (2/19)	SZ (34.1 ± 12.1) HC (33.7 ± 11.5)	PANSS	PS (17.4) NS (26.3)	11.5 years	3.0	NA	MNI	4
Ebdrup et al. (137)	SZ (38) HC (38)	SZ (10/28) HC (12/26)	SZ (25.9 ± 6.5) HC (25.8 ± 6.4)	PANSS	PS (20.7) NS (22.1)	75 weeks	3.0	NA	MNI	4
Xi et al. (19)	SZ (25) HC (25)	SZ (10/15) HC (9/16)	SZ (22.5 ± 4.3) HC (23.8 ± 3.0)	PANSS	PS (18.5) NS (20.4)	9.1 months	3.0	3.0	MNI	5

SZ, schizophrenia; HC, healthy control; GMV, gray matter volume; FA, fractional anisotropy; M/F, male/female; PANSS, Positive and Negative Syndrome Scale; SANS, Scale for the Assessment of Negative Symptom; NS, negative symptoms; PS: positive symptoms; MNI, Montreal Neurologic Institute.

**TABLE 2:** Demographic and clinical information for the functional MRI studies included in the meta-analysis.

Study	Subjects (*n*)	Gender (F/M)	Age (mean ± SD)	NS scale	PS/NS	Duration	Scanner	Thickness	Coordinates	Foci.no
**ALFF/fALFF**										
Hoptman et al. (138)	SZ (29) HC (26)	SZ (3/26) HC (7/19)	SZ (36.5 ± 11) HC (41.9 ± 10.9)	PANSS	PS (18.4) NS (20.2)	13.0 years	1.5	1.0	Talairach	SZ < HC:15 SZ > HC:8
Cui et al. (139)	SZ (15) HC (19)	SZ (7/8) HC (7/10)	SZ (22.53 ± 4.07) HC (23.79 ± 3.75)	PANSS	PS (17.93) NS (22.73)	10.2 months	3.0	4.0	MNI	SZ < HC:1 SZ > HC:2
Alonso-Solís et al. (140)	SZ (19) HC (20)	SZ (6/13) HC (7/13)	SZ (40.5 ± 8.9) HC (37.75 ± 7.4)	PANSS	PS (17.89) NS (21.47)	16.11 years	3.0	1.0	MNI	SZ < HC:2 SZ > HC:2
Salvador et al. (141)	SZ (116) HC (122)	SZ (35/81) HC (40/82)	SZ (36.76 ± 11.1) HC (36.51 ± 10.7)	PANSS	PS (16.57) NS (19.57)	14.88 years	1.5	7.0	MNI	SZ < HC:4 SZ > HC:6
Lian et al. (142)	SZ (18) HC (30)	SZ (10/8) HC (14/16)	SZ (20.44 ± 2.99) HC (20.53 ± 2.10)	PANSS	PS (19.13) NS (21.50)	7.89 months	3.0	1.0	MNI	SZ < HC:2 SZ > HC:4
Wu et al. (143)	SZ (32) HC (32)	SZ (16/16) HC (11/21)	SZ (30.94 ± 8.25) HC (31.37 ± 7.84)	PANSS	PS (20.0) NS (20.59)	8.91 months	3.0	4.0	MNI	SZ < HC:2 SZ > HC:6
**ReHo**										
Gao et al. (144)	SZ (14) HC (14)	SZ (5/9) HC (5/9)	SZ (33.2 ± 10.7) HC (34.9 ± 13.6)	PANSS	PS (16.4) NS (22.6)	9.2 years	1.5	5.0	MNI	SZ < HC:1 SZ > HC:1
Cui et al. (139)	SZ (15) HC (19)	SZ (7/8) HC (9/10)	SZ (22.53 ± 4.07) HC (23.79 ± 3.75)	PANSS	PS (17.93) NS (22.73)	10.2 months	3.0	4.0	MNI	SZ < HC:0 SZ > HC:2
Gou et al. (145)	SZ (28) HC (21)	SZ (12/16) HC (7/14)	SZ (23.9 ± 5.4) HC (28.8 ± 6.1)	PANSS	PS (17.8) NS (21.0)	15.1 months	1.5	5.0	MNI	SZ < HC:2 SZ > HC:0
Zhao et al. (28)	SZ (44) HC (26)	SZ (13/31) HC (9/17)	SZ (23.7 ± 5.3) HC (22.6 ± 3.7)	PANSS	PS (15.3) NS (24.7)	12.0 months	3.0	1.0	MNI	SZ < HC:5 SZ > HC:5
Yang et al. (146)	SZ (37) HC (39)	SZ (28/9) HC (30/9)	SZ (39.7 ± 10.84) HC (40.94 ± 6.27)	PANSS	PS (13.6) NS (23.9)	17.0 years	3.0	4.0	MNI	SZ < HC:5 SZ > HC:5
**FC**										
Bluhm et al. (147)	SZ (17) HC (17)	SZ (3/14) HC (3/14)	SZ (33.54 ± 13.7) HC (30.94 ± 12.6)	SANS	SAPS (9.06) SANS (23.9)	117.37 months	4.0	4.0	MNI	SZ < HC:8 SZ > HC:0
Fan et al. (148)	SZ (27) HC (15)	SZ (11/16) HC (8/7)	SZ (39.7 ± 7.2) HC (41.4 ± 6.3)	PANSS	PS (11.8) NS (19.9)	16.5 years	3.0	1.4	MNI	SZ < HC:3 SZ > HC:2
Chang et al. (149)	SZ (25) HC (25)	SZ (12/13) HC (10/15)	SZ (25.36 ± 6.32) HC (25.56 ± 6.78)	PANSS	PS (18.73) NS (21.39)	18.32 months	NA	NA	MNI	SZ < HC:5 SZ > HC:5
Manoliu et al. (150)	SZ (18) HC (20)	SZ (11/9) HC (9/9)	SZ (35.3 ± 12.5) HC (34 ± 13.35)	PANSS	PS (18.06) NS (19.94)	7.0 years	3.0	4.0	MNI	SZ < HC:14 SZ > HC:7
Zhuo et al. (151)	SZ (95) HC (93)	SZ (41/54) HC (48/45)	SZ (33.6 ± 7.8) HC (33 ± 10.2)	PANSS	PS (17.1) NS (20.3)	121.4 months	3.0	1.0	MNI	SZ < HC:4 SZ > HC:5
Alonso-Solís et al. (152)	SZ (19) HC (20)	SZ (6/13) HC (7/13)	SZ (40.05 ± 8.9) HC (37.75 ± 7.4)	PANSS	PS (17.89) NS (21.47)	16.11 years	3.0	1.0	MNI	SZ < HC:8 SZ > HC:14
Chang et al. (153)	SZ (18) HC (20)	SZ (9/9) HC (9/11)	SZ (22.67 ± 3.85) HC (23.43 ± 6.48)	PANSS	PS (18.61) NS (22.06)	12.44 months	3.0	1.0	MNI	SZ < HC:2 SZ > HC:0
Duan et al. (154)	SZ (28) HC (31)	SZ (10/18) HC (14/17)	SZ (36.5 ± 11.5) HC (35.2 ± 12.7)	PANSS	PS (16.54) NS (19.61)	12 years	3.0	4.0	MNI	SZ < HC:3 SZ > HC:2
Wang et al. (155)	SZ (94) HC (102)	SZ (42/52) HC (57/45)	SZ (33.6 ± 7.7) HC (33.4 ± 10.6)	PANSS	PS (16.6) NS (20.2)	120.1 months	3.0	1.0	MNI	SZ < HC:52 SZ > HC:7
Xu et al. (156)	SZ (66) HC (76)	SZ (28/38) HC (38/38)	SZ (33 ± 7.6) HC (33 ± 10.4)	PANSS	PS (17.0) NS (21.1)	114.0 months	3.0	1.0	MNI	SZ < HC:3 SZ > HC:0
Zhou et al. (157)	SZ (91) HC (100)	SZ (40/51) HC (55/45)	SZ (33.8 ± 7.7) HC (33.3 ± 10.5)	PANSS	PS (16.6) NS (20.0)	120.1 months	3.0	1.0	MNI	SZ < HC:10 SZ > HC:0
Chen et al. (158)	SZ (46) HC (46)	SZ (14/32) HC (22/24)	SZ (41.54 ± 8.86) HC (39.05 ± 6.99)	PANSS	PS (12.52) NS (20.61)	16.27 months	3.0	NA	MNI	SZ < HC:11 SZ > HC:4
Liu et al. (159)	SZ (95) HC (104)	SZ (42/53) HC (58/46)	SZ (34.1 ± 9.2) HC (33.8 ± 10.9)	PANSS	PS (16.83) NS (20.20)	123.0 months	3.0	1.0	MNI	SZ < HC:0 SZ > HC:6
Penner et al. (160)	SZ (24) HC (24)	SZ (3/21) HC (16/8)	SZ (23.2 ± 4.2) HC (23.8 ± 4.3)	SANS	SAPS (10.3) SANS (22.5)	13.7 months	3.0	NA	MNI	SZ < HC:28 SZ > HC:0
Peters et al. (161)	SZ (21) HC (21)	SZ (11/10) HC (11/10)	SZ (34.1 ± 12.3) HC (33.5 ± 12.9)	PANSS	PS (19.4) NS (21.14)	7.15 years	3.0	4.0	MNI	SZ < HC:7 SZ > HC:0
Zhuo et al. (162)	SZ (95) HC (93)	SZ (41/54) HC (48/45)	SZ (33.6 ± 7.8) HC (33.0 ± 10.2)	PANSS	PS (17.1) NS (20.3)	121.4 months	3.0	1.0	MNI	SZ < HC:5 SZ > HC:8
Ferri et al. (163)	SZ (183) HC (178)	SZ (46/137) HC (52/126)	SZ (38.7 ± 11.5) HC (37.7 ± 11.2)	SANS	SAPS (14.0) SANS (20.0)	17.23 years	3.0	NA	MNI	SZ < HC:9 SZ > HC:21
Penner et al. (164)	SZ (24) HC (24)	SZ (3/21) HC (12/12)	SZ (23.2 ± 4.2) HC (23.8 ± 4.3)	SANS	SAPS (10.3) SANS (22.5)	13.7 months	3.0	NA	MNI	SZ < HC:6 SZ > HC:1
Penner et al. (165)	SZ (24) HC (24)	SZ (3/21) HC (12/12)	SZ (23.2 ± 4.2) HC (23.8 ± 4.3)	SANS	SAPS (10.3) SANS (22.5)	13.7 months	3.0	NA	MNI	SZ < HC:27 SZ > HC:0
Sharma et al. (166)	SZ (34) HC (19)	SZ (12/22) HC (7/12)	SZ (29.3 ± 7.1) HC (31.5 ± 7.0)	SANS	SAPS (15.0) SANS (31.7)	194.71 weeks	3.0	1.0	MNI	SZ < HC:12 SZ > HC:0
Dong et al. (167)	SZ (96) HC (122)	SZ (30/66) HC (41/81)	SZ (39.8 ± 11.5) HC (38.0 ± 14.7)	PANSS	PS (13.44) NS (20.73)	15.1 years	3.0	4.0	MNI	SZ < HC:0 SZ > HC:19
Yasuda et al. (168)	SZ (111) HC (633)	SZ (50/61) HC (318/315)	SZ (34.3 ± 10.3) HC (34.1 ± 12.9)	PANSS	PS (19.6) NS (21.4)	11.0 years	1.5	1.4	MNI	SZ < HC:44 SZ > HC:42

SZ, schizophrenia; HC, healthy control; ALFF, amplitude of low-frequency fluctuation; fALFF, fractional amplitude of low-frequency fluctuation; ReHo, regional homogeneity; FC, functional connectivity; M/F, male/female; PANSS, Positive and Negative Syndrome Scale; SANS, Scale for the Assessment of Negative Symptom; NS, negative symptoms; PS, positive symptoms; MNI, Montreal Neurologic Institute.

### Meta-analysis results

**FIGURE 2:**
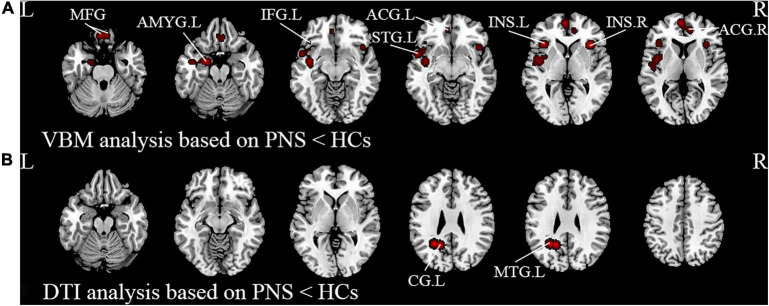
Results from the activation likelihood estimation (ALE) meta-analysis of structural abnormalities in schizophrenia with PNS. Brain regions showing **(A)** decreased GMV and **(B)** decreased FA in PNS patients compared with HCs. Significance threshold with a false discovery rate at *p* < 0.01. PNS, persistent negative symptoms; HCs, healthy controls; GMV, gray matter volume; FA, fractional anisotropy; VBM, voxel-based morphometry; DTI, diffusion tensor imaging; MFG, medial frontal gyrus; AMYG, amygdala; IFG, inferior frontal gyrus; STG, superior temporal gyrus; INS, insula; ACG, anterior cingulate gyrus; CG, cingulate gyrus; MTG, middle temporal gyrus; R, right; L, left.

Activation likelihood estimation (ALE) analysis indicated that schizophrenia with PNS, compared with HCs, showed significant GMV reductions in the bilateral insula, bilateral medial frontal gyrus (MFG), bilateral anterior cingulate gyrus (ACG), left amygdala, left superior temporal gyrus (STG), and left inferior frontal gyrus compared with HCs (Table 3 and Figure 2A). Analysis of the DTI studies revealed that schizophrenia patients with PNS showed reduced fractional anisotropy values in the left cingulate gyrus and middle temporal gyrus (Table 3 and Figure 2B).

**TABLE 3:** Brain structural and functional abnormalities in schizophrenia with PNS.

Cluster	Volume (mm^3^)	MNI			Peak ALE value	Brain regions	Side	BA
		
		*x*	*y*	*z*				
***GMV***								
**PNS < HC**								
1	12816	−36	22	0	0.025703	Insula	Left	13
1	12816	−20	−4	−20	0.02384	Amygdala	Left	*
1	12816	−40	−4	−10	0.0213	Insula	Left	13
1	12816	−52	8	−8	0.019044	Superior temporal gyrus	Left	22
1	12816	−44	−2	2	0.017317	Insula	Left	13
1	12816	−40	2	10	0.015336	Insula	Left	13
1	12816	−44	14	−6	0.014652	Insula	Left	13
1	12816	−42	−22	12	0.01359	Insula	Left	13
1	12816	−48	−12	8	0.011806	Insula	Left	13
1	12816	−42	26	−10	0.009091	Inferior frontal gyrus	Left	47
1	12816	−34	−6	−22	0.008715	Amygdala	Left	*
2	3296	−4	56	4	0.024632	Medial frontal gyrus	Left	10
2	3296	10	46	6	0.015118	Anterior cingulate gyrus	Right	32
3	3256	−4	36	−26	0.020289	Medial frontal gyrus	Left	11
3	3256	6	36	−26	0.017226	Medial frontal gyrus	Right	11
3	3256	4	34	−16	0.01442	Anterior cingulate gyrus	Right	32
3	3256	−6	44	−8	0.012126	Anterior cingulate gyrus	Left	32
4	2960	36	22	0	0.032923	Insula	Right	13
4	2960	44	18	−6	0.017102	Insula	Right	13
***FA***								
**PNS < HC**								
1	3928	−18	−54	28	0.009544	Cingulate gyrus	Left	31
1	3928	−27	−52	29	0.008892	Middle temporal gyrus	Left	39
***ALFF/fALFF***								
**PNS < HC**								
1	2536	−6	−70	18	0.01177	Posterior cingulate gyrus	Left	31
***ReHo***								
**PNS > HC**								
1	4832	−18	8	−4	0.010073	Lentiform nucleus	Left	*
1	4832	−18	8	−12	0.009689	Lentiform nucleus	Left	*
1	4832	−26	0	0	0.007537	Lentiform nucleus	Left	*
***FC***								
**PNS < HC**								
1	4744	−2	4	46	0.016104	Cingulate gyrus	Left	24
1	4744	−4	4	42	0.015914	Cingulate gyrus	Left	24
1	4744	0	0	50	0.015846	Medial frontal gyrus	Left	6
1	4744	4	18	42	0.012946	Cingulate gyrus	Right	32
1	4744	6	12	34	0.012707	Cingulate gyrus	Right	24
1	4744	10	12	40	0.012509	Cingulate gyrus	Right	32
1	4744	2	14	50	0.012247	Superior frontal gyrus	Left	6
1	4744	−6	0	62	0.011075	Medial frontal gyrus	Left	6
1	4744	0	18	30	0.010968	Cingulate gyrus	Left	24
1	4744	−2	12	34	0.010758	Cingulate gyrus	Left	24
1	4744	8	6	40	0.010547	Cingulate gyrus	Right	24
**PNS > HC**								
1	2344	6	−66	38	0.012566	Cuneus	Right	7
1	2344	6	−66	28	0.012217	Precuneus	Right	31
1	2344	−2	−70	40	0.011512	Precuneus	Left	7
1	2344	12	−66	36	0.010896	Cuneus	Right	7
1	2344	6	−66	18	0.010553	Posterior cingulate gyrus	Right	31
1	2344	6	−66	22	0.010255	Precuneus	Right	31
1	2344	12	−72	28	0.01018	Precuneus	Right	31
1	2344	6	−62	18	0.010163	Posterior cingulate gyrus	Right	23
2	2144	8	−26	−2	0.017002	Thalamus	Right	*
2	2144	8	−24	8	0.013201	Thalamus	Right	*
2	2144	−6	−28	0	0.010496	Thalamus	Left	*

BA, Brodmann Area; ALE, anatomical/activation likelihood estimation; MNI, Montreal Neurologic Institute; PNS, persistent negative symptoms; HC, healthy control; GMV, gray matter volume; FA, fractional anisotropy; ALFF, amplitude of low-frequency fluctuation; fALFF, fractional amplitude of low-frequency fluctuation; ReHo, regional homogeneity; FC, functional connectivity. *The peak coordinate is not at the Brodmann Area.

**FIGURE 3:**
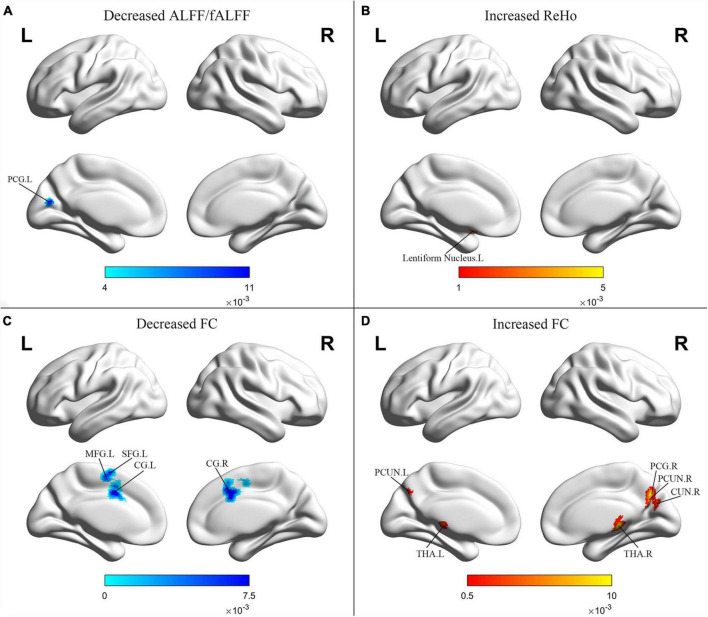
Results from the activation likelihood estimation (ALE) meta-analysis of functional abnormalities in schizophrenia with PNS. Brain regions showing **(A)** decreased ALFF/fALFF; **(B)** increased ReHo; **(C)** decreased FC; **(D)** increased FC, in schizophrenia with PNS compared with HCs. Significance threshold with a false discovery rate at *p* < 0.01. PNS, persistent negative symptoms; HCs, healthy controls; ALFF, amplitude of low-frequency fluctuation; fALFF, fractional amplitude of low-frequency fluctuation; ReHo, regional homogeneity; FC, functional connectivity; PCG, posterior cingulate gyrus; MFG, medial frontal gyrus; SFG, superior frontal gyrus; CG, cingulate gyrus; PCUN, precuneus; THA, thalamus; CUN, cuneus; R, right; L, left.

Schizophrenia patients with PNS exhibited decreased ALFF/fALFF in the left posterior cingulate gyrus (PCG). Additionally, these patients also showed increased ReHo in the left lentiform nucleus and decreased FC in the bilateral cingulate gyrus, left MFG and left superior frontal gyrus. Compared to HCs, PNS patients presented increased FC in the bilateral precuneus, bilateral thalamus, right cuneus and right PCG (Table 3 and Figure 3).

## Discussion

To the best of our knowledge, this is the first meta-analysis of whole-brain structural and functional MRI findings for schizophrenia with PNS. Specifically, the main findings of the present article comprise: (1) decreased GMV in bilateral insula, bilateral MFG, bilateral ACG, left amygdala, left STG, and left inferior frontal gyrus in the PNS group; (2) reduced FA values in the left cingulate gyrus and middle temporal gyrus; (3) increased ReHo in the left lentiform nucleus and enhanced FC in the bilateral precuneus, bilateral thalamus, right cuneus, right PCG; and (4) decreased ALFF/fALFF in the left PCG and reduced FC in the bilateral cingulate gyrus, left MFG and left superior frontal gyrus.

### Structural magnetic resonance imaging results

In the present study, the VBM meta-analysis results revealed reduced GMV in the prefrontal gyrus and ACG in schizophrenia with PNS compared to HCs. Several neuroimaging studies have reported that decreased GMV in the prefrontal gyrus was negatively correlated with negative symptom severity (53, 54), which might be related to impaired self-reference processing and social cognition (55). Our findings are in line with a previous study that reported a negative correlation between reduced GMV in the ACG and negative symptoms in schizophrenia patients (56) as well as a study that showed right hemispheric ACG volume reduction in schizophrenia with PNS compared to HCs (57). It was previously demonstrated that the medial frontal lobe wall, composed of the ACG and medial prefrontal gyrus (58), plays a key role in social cognitive processing, particularly in mentalizing others’ intentions (59), thereby suggesting that abnormalities in this region could lead to difficulties in interacting with others. Consequently, we speculate that the reduced GMV in the medial frontal lobe wall might underlie the increasing social withdrawal that is characteristic of negative symptoms. However, these findings were not replicated in other studies (60, 61). These inconsistent results might be attributed to variable criteria in defining PNS as well as the heterogeneity of schizophrenia, including disease courses, and the use of antipsychotics.

In our meta-analysis, apart from the frontal gyrus, subcortical regions such as the insula and amygdala have also shown reduced GMV in schizophrenia with PNS. The insula plays a key role in monitoring internal emotional states (62) and regulating the influences of emotion on cognitive processes (63, 64). Many studies have reported abnormalities in the insula which were related to negative symptoms in schizophrenia (17, 65). These are in accordance with findings that a volumetric decrease of the insula relative to controls could be detected in schizophrenia with PNS (66, 67). Similarly, structural abnormalities in the left amygdala were found to be significantly associated with PANSS negative symptoms (68), which is consistent with studies that found a negative correlation between the volume of the hippocampus-amygdala complex and clinical ratings of negative symptoms and thought disturbances (69, 70). Nevertheless, the increased GMV in the amygdala and its negative correlation with PANSS scores have also been reported (71). Considering the function of the amygdala in regulating emotional and motivational behavior (72), it is reasonable that there is an association between the amygdala and negative symptoms of reduced expression in schizophrenia. Together, these findings might explain the various symptom profiles of patients and psychopathology, such as the loss of boundaries, lack of emotional reactivity, and poor empathy.

Alterations of the temporal lobe in schizophrenia have been investigated by a considerable number of neuroimaging studies. We also observed a significant decrease in the GMV of the STG in patients with PNS. The STG is involved in emotion processing, particularly negative emotions as shown in studies of facial emotion perception (73, 74). Previous articles reported that schizophrenia patients had significantly smaller bilateral STG volumes than HCs (75), which was negatively correlated with the severity of auditory hallucinations and thought disorder (76, 77). Consistent with our results, one study of schizophrenia with PNS found a reduction of gray matter in the left STG (78). Several VBM analyses found that the PNS patients showed more prominent and extended alterations affecting the prefrontal, temporal, limbic and subcortical regions compared to the non-PNS patients (15, 79). Altogether, these findings suggest that smaller GMV in these regions appear to be a substrate for schizophrenia with PNS. It remains to be seen whether these regions contribute directly to the pathophysiological process of patients with PNS.

White matter abnormalities have long been reported in schizophrenia patients with inconsistent results (80, 81), and the correlation between the negative symptoms and white matter defects has also been confirmed (82–84). The present meta-analysis additionally observed decreased FA in the left cingulate gyrus and middle temporal gyrus in schizophrenia with PNS. The cingulate cortex is a critical region in the saliency and cognitive motor circuit, with the ACG involved in the decision-making circuit and emotional processing (85). It has been previously reported that there is a significant association between reduced FA in the ACG and avolition-apathy and anhedonia in schizophrenia (86, 87). The middle temporal gyrus is a critical component of the neural network involved in pleasure and reward (88). Our results align with previous reports of FA deficits in the deep temporal lobe in patients with PNS (89, 90). Hence, the decreased white matter FA in the middle temporal lobe might reflect impairments in reward-related processing in schizophrenia with PNS.

### Functional magnetic resonance imaging results

The finding of decreased ALFF/fALFF in the left PCG in schizophrenia with PNS is consistent with previous data demonstrating a negative correlation of ALFF in the left PCG with negative symptoms and withdrawal on the PANSS (91). These observations are in accordance with the notion that a dysregulation between the striatum and PCG is associated with cognitive-affective control (92), which might provide a neurophysiological basis for negative symptoms. ReHo abnormalities were also detected in the lentiform nucleus that is involved in the basal ganglia-thalamocortical circuitry (93). Nevertheless, an increased ReHo in the lentiform nucleus in patients with PNS is seldom reported and might represent a protective or compensatory phenomenon. One study indicated that increased ReHo in the lentiform nucleus was not related to negative symptoms (28), while other studies found that increased ReHo values in the right inferior frontal gyrus/insula may reflect the severity of negative symptoms and verbal learning abilities (34). However, in our study, we did not observe consistent results from the ALE analysis. This could be explained by the fact that there exist limited functional MRI studies investigating ReHo changes in PNS patients.

In this study, a decreased FC was detected in the MFG and superior frontal gyrus whilst an increased FC was found in the right PCG and bilateral precuneus. Interestingly, these areas overlap with the DMN, which is involved in the processing of task-independent thoughts, attention to internal emotional states, self-inspection, and future planning (36, 94). Decreased connectivity in the DMN was observed in previous studies (95, 96), and related to clinical symptoms and cognitive performance (97, 98). Although several studies have reported that the DMN connectivity in the prefrontal cortices correlated negatively with the severity of positive and mood symptoms in patients with schizophrenia (99), the connectivity between the prefrontal cortices and PCG was differentially related to social attainment and social competence (100). The results of the present meta-analysis showed hyperconnectivity (right PCG and bilateral precuneus) as well as hypoconnectivity (MFG and superior frontal gyrus) in the DMN. These findings are in line with other recent studies which indicated associations between high DMN resting-state connectivity and negative symptoms in schizophrenia patients (101, 102). Previous evidence has also suggested that the transition probability from a state with weak precuneus/PCG connectivity to stronger connectivity increased with symptom severity (103), thereby demonstrating the functional significance of the relationship between negative symptoms and increased DMN connectivity in schizophrenia. These results suggest that the DMN is often hyperconnected in schizophrenia with PNS, which and might be related to the overly intense self-reference and impairments in attention and working memory observed in these patients (104, 105).

In our study, increased FC was mainly observed in the thalamo-cortical network, including the bilateral thalamus, bilateral precuneus, and right PCG. The thalamus, which is involved in a great variety of cognitive functions and mental activities including memory, language, perception and emotion, represents a key node in distributed neuronal circuits involving various regions of the cerebral cortex, striatum and cerebellum (106–109). Individuals at high clinical risk for psychosis have enhanced connectivity in cerebellar-thalamo-cortical circuits which was significantly associated with positive symptoms (110). Similar findings were also found in patients with schizophrenia through a study of an independent clinical sample (111). Consistent with our findings, Anticevic et al. reported a positive correlation between schizophrenia total symptom severity and all regions displaying hyperconnectivity with the thalamus (112). In a recent study, higher cerebello-thalamo-cortical connectivity at baseline significantly predicted poorer long-term reduction in negative symptoms (113). Numerous functional MRI studies have also reported reduced thalamic-prefrontal connectivity and increased coupling with somatomotor and temporal regions in schizophrenia (114–116). These findings support the theory that thalamo-cortical interactions are critical for optimal brain functioning and provide further evidence for the role of thalamo-cortical interactions in the pathophysiology of schizophrenia.

### Clinical implications

The present findings have a few implications for our understanding of both the neural mechanisms of PNS patients and the development of the intervention. Firstly, altered GMV in the prefrontal, temporal, limbic, and subcortical regions might be the key anatomical basis for PNS since these regions were consistently identified in different meta-analyses. Moreover, patients with PNS can benefit from more thorough assessment with multiple imaging techniques, as these data can help researchers to design individualized interventions to achieve better treatment outcomes. Taken together, our findings reveal provide evidence of the specificity of the affected brain regions and provide new insights for targeted treatment and follow-up care.

## Limitations

The present study has several limitations. Firstly, due to different terms and definitions of PNS, the included studies did not fully conform to the PNS criteria proposed by Buchanan (3). The assessment of negative symptoms of patients with PNS by different scales may lead to heterogeneity of results. Secondly, the ALE methodology we used had certain limitations. For example, the ALE software could not analyze the correlation between the severity of negative symptoms and these brain regions, and it failed to provide any solving approach to analyze the confidence interval to increase the robustness of our findings. Thirdly, the literature on whole-brain ALFF, ReHo, and DTI data in schizophrenia patients with PNS is very limited, and the small sample size of available articles weakens the validity of our meta-analysis. Next, there was substantial heterogeneity among patients with PNS, including time to the first episode, antipsychotic medication, and duration of negative symptoms. Another inherent limitation of this meta-analysis approach is the heterogeneity of the results, which might arise from differences in methodology across studies, including imaging acquisition and analysis pipelines, clinical assessments, and small sample sizes.

## Conclusion

By performing ALE meta-analysis in PNS patients to identify structural and functional alterations, we found that structural brain abnormalities were consistently located in the insula, medial and inferior frontal gyrus, anterior cingulate gyrus, amygdala, superior temporal gyrus and middle temporal gyrus, and functional alterations were concentrated in the thalamo-cortical circuits and the DMN. In addition, we observed that enhanced functional alterations were detected in thalamo-cortical circuits in patients with PNS, thereby demonstrating that it plays an important role in the diagnosis and prediction of negative symptoms in schizophrenia. These findings help to elucidate the brain abnormalities specific to schizophrenia patients with PNS, which are important for understanding their underlying the pathophysiology and may ultimately contribute to the development of future behavioral, pharmacological, or neurotherapeutic treatments.

## Data availability statement

The original contributions presented in this study are included in the article/Supplementary material, further inquiries can be directed to the corresponding authors.

## Author contributions

TZ guided by XZ and JC designed the study. TZ and ZW performed the meta-analysis and drafted the manuscript. CZ, XF, CH, CX, HG, and ZY helped in literature extraction and data analyses. All authors contributed to the article and approved the submitted version.

## Footnotes

1
http://brainmap.org/ale/

2
http://ric.uthscsa.edu/mango

3
http://rfmri.org/dpabi
