# Supplementary material for: Meta-analysis of structural and functional brain abnormalities in schizophrenia with persistent negative symptoms using activation likelihood estimation
Source: Front Psychiatry. 2022 Sep 27;13:957685. doi: 10.3389/fpsyt.2022.957685 (PMC9552970; doi:10.3389/fpsyt.2022.957685)
Supplement: Supplementary file 1 [file Table_1.docx]

**Table S1. The checklist of imaging methodology quality assessment for all the articles included in the present meta-analysis.**

| **Study** | **Category 1: Subjects** | | | | **Category 2: Methods for image acquisition and analysis** | | | | | | **Category 3: Results and conclusions** | |  |
| --- | --- | --- | --- | --- | --- | --- | --- | --- | --- | --- | --- | --- | --- |
|  | Patients were evaluated prospectively, specific diagnostic criteria were applied, and demographic data were reported | Healthy comparison participants were evaluated prospectively; psychiatric and medical illnesses were excluded | Important variables (e.g., age, gender, drug status, illness duration, and symptom severity) were checked either via stratification or statistics | Sample size per group: ≥ 20, scores 1; ≥ 10, scores 0.5 | All neuroanatomic measurements were made blind to group assignment and to subjects’ identity | Measures for brain structures were reported | Magnet strength: 3T, scores 1; 1.5T, scores 0.5 | The imaging technique used was clearly described so that it could be reproduced | Whole brain analysis was automated without a previously defined region | Spatial coordinates were reported in a standard space (e.g., Talairach or MNI coordinates) | Statistical results were corrected for multiple comparison scores 1, uncorrected scores 0.5 | Conclusions were consistent with the results obtained, and the limitations were discussed | total |
| **GMV** | | | | | | | | | | | | | |
| Paillère-Martinot^[1]^ | 1 | 1 | 1 | 1 | 1 | 1 | 0.5 | 1 | 1 | 1 | 0.5 | 1 | 11 |
| Sigmundsson^[2]^ | 1 | 1 | 0.5 | 1 | 1 | 1 | 0.5 | 1 | 1 | 1 | 0.5 | 1 | 10.5 |
| Kawasaki^[3]^ | 1 | 1 | 0.5 | 1 | 1 | 1 | 0.5 | 1 | 1 | 1 | 1 | 1 | 11 |
| Jayakumar^[4]^ | 1 | 1 | 1 | 0.5 | 1 | 1 | 0.5 | 1 | 1 | 1 | 1 | 1 | 11 |
| Bassitt^[5]^ | 1 | 1 | 0.5 | 1 | 1 | 1 | 0.5 | 1 | 1 | 1 | 1 | 1 | 11 |
| Koutsouleris^[6]^ | 1 | 1 | 0.5 | 1 | 1 | 1 | 0.5 | 1 | 1 | 1 | 1 | 1 | 11 |
| Meisenzahl^[7]^ | 1 | 1 | 0.5 | 1 | 1 | 1 | 0.5 | 1 | 1 | 1 | 1 | 1 | 11 |
| Herold^[8]^ | 1 | 1 | 1 | 0.5 | 1 | 1 | 0.5 | 1 | 1 | 1 | 0.5 | 1 | 10.5 |
| Whitford^[9]^ | 1 | 1 | 0.5 | 1 | 1 | 1 | 0.5 | 1 | 1 | 1 | 1 | 1 | 11 |
| Cascella^[10]^ | 1 | 1 | 0.5 | 0.5 | 1 | 1 | 0.5 | 1 | 1 | 1 | 1 | 1 | 10.5 |
| Anderson^[11]^ | 1 | 1 | 0.5 | 0.5 | 1 | 1 | 1 | 1 | 1 | 1 | 1 | 1 | 11 |
| Huang,P^[12]^ | 1 | 1 | 0.5 | 0.5 | 1 | 1 | 1 | 1 | 1 | 1 | 0.5 | 1 | 10.5 |
| Poletti^[13]^ | 1 | 1 | 0.5 | 1 | 1 | 1 | 1 | 1 | 1 | 1 | 1 | 1 | 11.5 |
| Huang,X^[14]^ | 1 | 1 | 1 | 1 | 1 | 1 | 1 | 1 | 1 | 1 | 1 | 1 | 12 |
| Kim,G. W^[15]^ | 1 | 1 | 1 | 1 | 1 | 1 | 1 | 1 | 1 | 1 | 1 | 1 | 12 |
| Kuroki^[16]^ | 1 | 1 | 0.5 | 0.5 | 1 | 1 | 1 | 1 | 1 | 1 | 1 | 1 | 11 |
| Szendi^[17]^ | 1 | 1 | 1 | 0.5 | 1 | 1 | 0.5 | 1 | 1 | 1 | 1 | 1 | 11 |
| Spalthoff^[18]^ | 1 | 1 | 1 | 1 | 1 | 1 | 1 | 1 | 1 | 1 | 1 | 1 | 12 |
| Zhao^[19]^ | 1 | 1 | 0.5 | 1 | 1 | 1 | 1 | 1 | 1 | 1 | 0.5 | 1 | 11 |
| Lei^[20]^ | 1 | 1 | 1 | 0.5 | 1 | 1 | 1 | 1 | 1 | 1 | 1 | 1 | 11.5 |
| Neugebauer^[21]^ | 1 | 1 | 1 | 0.5 | 1 | 1 | 1 | 1 | 1 | 1 | 1 | 1 | 11.5 |
| **FA** | | | | | | | | | | | | | |
| Rametti^[22]^ | 1 | 1 | 1 | 1 | 1 | 1 | 0.5 | 1 | 1 | 1 | 1 | 1 | 11.5 |
| Spalletta^[23]^ | 1 | 1 | 0.5 | 1 | 1 | 1 | 1 | 1 | 1 | 1 | 1 | 1 | 11.5 |
| Ebdrup^[24]^ | 1 | 1 | 0.5 | 1 | 1 | 1 | 1 | 1 | 1 | 1 | 1 | 1 | 11.5 |
| Xi^[25]^ | 1 | 1 | 0.5 | 1 | 1 | 1 | 1 | 1 | 1 | 1 | 1 | 1 | 11.5 |
| **ALFF/fALFF** | | | | | | | | | | | | | |
| Hoptman^[26]^ | 1 | 1 | 0.5 | 1 | 1 | 1 | 0.5 | 1 | 1 | 1 | 0.5 | 1 | 10.5 |
| Cui,L^[27]^ | 1 | 1 | 1 | 0.5 | 1 | 1 | 1 | 1 | 1 | 1 | 1 | 1 | 11.5 |
| Alonso-Solís^[28]^ | 1 | 1 | 1 | 0.5 | 1 | 1 | 1 | 1 | 1 | 1 | 1 | 1 | 11.5 |
| Salvador^[29]^ | 1 | 1 | 0.5 | 1 | 1 | 1 | 0.5 | 1 | 1 | 1 | 1 | 1 | 11 |
| Lian,N^[30]^ | 1 | 1 | 1 | 0.5 | 1 | 1 | 1 | 1 | 1 | 1 | 1 | 1 | 11.5 |
| Wu,R^[31]^ | 1 | 1 | 1 | 1 | 1 | 1 | 1 | 1 | 1 | 1 | 1 | 1 | 12 |
| **ReHo** | | | | | | | | | | | | | |
| Gao,B^[32]^ | 1 | 1 | 1 | 0.5 | 1 | 1 | 0.5 | 1 | 1 | 1 | 1 | 1 | 11 |
| Cui,L.B^[27]^ | 1 | 1 | 1 | 0.5 | 1 | 1 | 1 | 1 | 1 | 1 | 1 | 1 | 11.5 |
| Gou,N^[33]^ | 1 | 1 | 0.5 | 1 | 1 | 1 | 0.5 | 1 | 1 | 1 | 1 | 1 | 11 |
| Zhao,X^[34]^ | 1 | 1 | 1 | 1 | 1 | 1 | 1 | 1 | 1 | 1 | 1 | 1 | 12 |
| Yang,F^[35]^ | 1 | 1 | 0.5 | 1 | 1 | 1 | 1 | 1 | 1 | 1 | 1 | 1 | 11.5 |
| **FC** | | | | | | | | | | | | | |
| Bluhm^[36]^ | 1 | 1 | 0.5 | 0.5 | 1 | 1 | 1 | 1 | 1 | 1 | 0.5 | 1 | 10.5 |
| Fan,F.M^[37]^ | 1 | 1 | 0.5 | 1 | 1 | 1 | 1 | 1 | 1 | 1 | 1 | 1 | 11.5 |
| Chang,X^[38]^ | 1 | 1 | 1 | 1 | 1 | 1 | 1 | 1 | 1 | 1 | 1 | 1 | 12 |
| Manoliu^[39]^ | 1 | 1 | 0.5 | 0.5 | 1 | 1 | 1 | 1 | 1 | 1 | 0.5 | 1 | 10.5 |
| Zhuo,C^[40]^ | 1 | 1 | 0.5 | 1 | 1 | 1 | 1 | 1 | 1 | 1 | 1 | 1 | 11.5 |
| Alonso-Solís^[41]^ | 1 | 1 | 0.5 | 0.5 | 1 | 1 | 1 | 1 | 1 | 1 | 1 | 1 | 11 |
| Chang,X^[42]^ | 1 | 1 | 1 | 0.5 | 1 | 1 | 0.5 | 1 | 1 | 1 | 1 | 1 | 11 |
| Duan,M^[43]^ | 1 | 1 | 1 | 1 | 1 | 1 | 1 | 1 | 1 | 1 | 1 | 1 | 12 |
| Wang,D^[44]^ | 1 | 1 | 0.5 | 1 | 1 | 1 | 1 | 1 | 1 | 1 | 1 | 1 | 11.5 |
| Xu,L^[45]^ | 1 | 1 | 0.5 | 1 | 1 | 1 | 1 | 1 | 1 | 1 | 1 | 1 | 11.5 |
| Zhou,Y^[46]^ | 1 | 1 | 0.5 | 1 | 1 | 1 | 1 | 1 | 1 | 1 | 1 | 1 | 11.5 |
| Chen,X^[47]^ | 1 | 1 | 1 | 1 | 1 | 1 | 1 | 1 | 1 | 1 | 1 | 1 | 12 |
| Liu, X^[48]^ | 1 | 1 | 0.5 | 1 | 1 | 1 | 1 | 1 | 1 | 1 | 0.5 | 1 | 11 |
| Penner,J^[49]^ | 1 | 1 | 1 | 1 | 1 | 1 | 1 | 1 | 1 | 1 | 1 | 1 | 12 |
| Peters^[50]^ | 1 | 1 | 0.5 | 1 | 1 | 1 | 1 | 1 | 1 | 1 | 1 | 1 | 11.5 |
| Zhuo,C^[51]^ | 1 | 1 | 0.5 | 1 | 1 | 1 | 1 | 1 | 1 | 1 | 1 | 1 | 11.5 |
| Ferri.^[52]^ | 1 |  | 0.5 | 1 | 1 | 1 | 1 | 1 | 1 | 1 | 0.5 | 1 | 11 |
| Penner,J.a^[53]^ | 1 | 1 | 0.5 | 1 | 1 | 1 | 1 | 1 | 1 | 1 | 1 | 1 | 11.5 |
| Penner,J.b^[54]^ | 1 | 1 | 0.5 | 1 | 1 | 1 | 1 | 1 | 1 | 1 | 1 | 1 | 11.5 |
| Sharma^[55]^ | 1 | 1 | 0.5 | 1 | 1 | 1 | 1 | 1 | 1 | 1 | 1 | 1 | 11.5 |
| Dong^[56]^ | 1 | 1 | 1 | 1 | 1 | 1 | 1 | 1 | 1 | 1 | 1 | 1 | 12 |
| Yasuda^[57]^ | 1 | 1 | 0.5 | 1 | 1 | 1 | 0.5 | 1 | 1 | 1 | 1 | 1 | 11 |

1. Paillère-Martinot, M., et al., *Cerebral gray and white matter reductions and clinical correlates in patients with early onset schizophrenia.* Schizophr Res, 2001. **50**(1-2): p. 19-26.

2. Sigmundsson, T., et al., *Structural abnormalities in frontal, temporal, and limbic regions and interconnecting white matter tracts in schizophrenic patients with prominent negative symptoms.* Am J Psychiatry, 2001. **158**(2): p. 234-43.

3. Kawasaki, Y., et al., *Structural brain differences in patients with schizophrenia and schizotypal disorder demonstrated by voxel-based morphometry.* Eur Arch Psychiatry Clin Neurosci, 2004. **254**(6): p. 406-14.

4. Jayakumar, P.N., et al., *Optimized voxel-based morphometry of gray matter volume in first-episode, antipsychotic-naive schizophrenia.* Prog Neuropsychopharmacol Biol Psychiatry, 2005. **29**(4): p. 587-91.

5. Bassitt, D.P., et al., *Insight and regional brain volumes in schizophrenia.* Eur Arch Psychiatry Clin Neurosci, 2007. **257**(1): p. 58-62.

6. Koutsouleris, N., et al., *Structural correlates of psychopathological symptom dimensions in schizophrenia: a voxel-based morphometric study.* Neuroimage, 2008. **39**(4): p. 1600-12.

7. Meisenzahl, E.M., et al., *Structural brain alterations at different stages of schizophrenia: A voxel-based morphometric study.* Schizophrenia Research, 2008. **104**(1-3): p. 44-60.

8. Herold, R., et al., *Regional gray matter reduction and theory of mind deficit in the early phase of schizophrenia: a voxel-based morphometric study.* Acta Psychiatr Scand, 2009. **119**(3): p. 199-208.

9. Whitford, T.J., et al., *Delusions and dorso-medial frontal cortex volume in first-episode schizophrenia: A voxel-based morphometry study.* Psychiatry Research-Neuroimaging, 2009. **172**(3): p. 175-179.

10. Cascella, N.G., et al., *Gray-matter abnormalities in deficit schizophrenia.* Schizophr Res, 2010. **120**(1-3): p. 63-70.

11. Anderson, V.M., et al., *Extensive Gray Matter Volume Reduction in Treatment-Resistant Schizophrenia.* International Journal of Neuropsychopharmacology, 2015. **18**(7).

12. Huang, P., et al., *Decreased bilateral thalamic gray matter volume in first-episode schizophrenia with prominent hallucinatory symptoms: A volumetric MRI study.* Sci Rep, 2015. **5**: p. 14505.

13. Poletti, S., et al., *Adverse childhood experiences influence the detrimental effect of bipolar disorder and schizophrenia on cortico-limbic grey matter volumes.* J Affect Disord, 2016. **189**: p. 290-7.

14. Huang, X., et al., *Decreased Left Putamen and Thalamus Volume Correlates with Delusions in First-Episode Schizophrenia Patients.* Front Psychiatry, 2017. **8**: p. 245.

15. Kim, G.W., Y.H. Kim, and G.W. Jeong, *Whole brain volume changes and its correlation with clinical symptom severity in patients with schizophrenia: A DARTEL-based VBM study.* PLoS One, 2017. **12**(5): p. e0177251.

16. Kuroki, N., et al., *Brain structure differences among male schizophrenic patients with history of serious violent acts: an MRI voxel-based morphometric study.* BMC Psychiatry, 2017. **17**(1): p. 105.

17. Szendi, I., et al., *A New Division of Schizophrenia Revealed Expanded Bilateral Brain Structural Abnormalities of the Association Cortices.* Front Psychiatry, 2017. **8**: p. 127.

18. Spalthoff, R., C. Gaser, and I. Nenadić, *Altered gyrification in schizophrenia and its relation to other morphometric markers.* Schizophr Res, 2018. **202**: p. 195-202.

19. Zhao, C.a., et al., *Structural and functional brain abnormalities in schizophrenia: A cross-sectional study at different stages of the disease.* Progress in Neuro Psychopharmacology & Biological Psychiatry, 2018. **83**: p. 27-32.

20. Lei, W., et al., *Progressive brain structural changes after the first year of treatment in first-episode treatment-naive patients with deficit or nondeficit schizophrenia.* Psychiatry Res Neuroimaging, 2019. **288**: p. 12-20.

21. Neugebauer, K., et al., *Nerve Growth Factor Serum Levels Are Associated With Regional Gray Matter Volume Differences in Schizophrenia Patients.* Front Psychiatry, 2019. **10**: p. 275.

22. Rametti, G., et al., *A voxel-based diffusion tensor imaging study of temporal white matter in patients with schizophrenia.* Psychiatry Res, 2009. **171**(3): p. 166-76.

23. Spalletta, G., et al., *Brain white matter microstructure in deficit and non-deficit subtypes of schizophrenia.* Psychiatry Res, 2015. **231**(3): p. 252-61.

24. Ebdrup, B.H., et al., *Frontal fasciculi and psychotic symptoms in antipsychotic-naive patients with schizophrenia before and after 6 weeks of selective dopamine D2/3 receptor blockade.* J Psychiatry Neurosci, 2016. **41**(2): p. 133-41.

25. Xi, Y.B., et al., *The structural connectivity pathology of first-episode schizophrenia based on the cardinal symptom of auditory verbal hallucinations.* Psychiatry Res Neuroimaging, 2016. **257**: p. 25-30.

26. Hoptman, M.J., et al., *Amplitude of low-frequency oscillations in schizophrenia: a resting state fMRI study.* Schizophr Res, 2010. **117**(1): p. 13-20.

27. Cui, L.B., et al., *Putamen-related regional and network functional deficits in first-episode schizophrenia with auditory verbal hallucinations.* Schizophr Res, 2016. **173**(1-2): p. 13-22.

28. Alonso-Solís, A., et al., *Altered amplitude of low frequency fluctuations in schizophrenia patients with persistent auditory verbal hallucinations.* Schizophr Res, 2017. **189**: p. 97-103.

29. Salvador, R., et al., *Non redundant functional brain connectivity in schizophrenia.* Brain Imaging and Behavior, 2017. **11**(2): p. 552-564.

30. Lian, N., et al., *A comparative study of magnetic resonance imaging on the gray matter and resting-state function in prodromal and first-episode schizophrenia.* Am J Med Genet B Neuropsychiatr Genet, 2018. **177**(6): p. 537-545.

31. Wu, R., et al., *Reduced Brain Activity in the Right Putamen as an Early Predictor for Treatment Response in Drug-Naive, First-Episode Schizophrenia.* Front Psychiatry, 2019. **10**: p. 741.

32. Gao, B., et al., *Spontaneous Activity Associated with Delusions of Schizophrenia in the Left Medial Superior Frontal Gyrus: A Resting-State fMRI Study.* PLoS One, 2015. **10**(7): p. e0133766.

33. Gou, N., et al., *Effects of DISC1 Polymorphisms on Resting-State Spontaneous Neuronal Activity in the Early-Stage of Schizophrenia.* Front Psychiatry, 2018. **9**: p. 137.

34. Zhao, X., et al., *Abnormalities of regional homogeneity and its correlation with clinical symptoms in Naïve patients with first-episode schizophrenia.* Brain Imaging Behav, 2019. **13**(2): p. 503-513.

35. Yang, F., et al., *Correlation of abnormalities in resting state fMRI with executive functioning in chronic schizophrenia.* Psychiatry Res, 2021. **299**: p. 113862.

36. Bluhm, R.L., et al., *Spontaneous low-frequency fluctuations in the BOLD signal in schizophrenic patients: anomalies in the default network.* Schizophr Bull, 2007. **33**(4): p. 1004-12.

37. Fan, F.M., et al., *Ventral medial prefrontal functional connectivity and emotion regulation in chronic schizophrenia: a pilot study.* Neurosci Bull, 2013. **29**(1): p. 59-74.

38. Chang, X., et al., *Altered default mode and fronto-parietal network subsystems in patients with schizophrenia and their unaffected siblings.* Brain Res, 2014. **1562**: p. 87-99.

39. Manoliu, A., et al., *Aberrant dependence of default mode/central executive network interactions on anterior insular salience network activity in schizophrenia.* Schizophr Bull, 2014. **40**(2): p. 428-37.

40. Zhuo, C., et al., *Functional connectivity density alterations in schizophrenia.* Front Behav Neurosci, 2014. **8**: p. 404.

41. Alonso-Solís, A., et al., *Resting-state functional connectivity alterations in the default network of schizophrenia patients with persistent auditory verbal hallucinations.* Schizophr Res, 2015. **161**(2-3): p. 261-8.

42. Chang, X., et al., *Distinct inter-hemispheric dysconnectivity in schizophrenia patients with and without auditory verbal hallucinations.* Sci Rep, 2015. **5**: p. 11218.

43. Duan, M., et al., *Altered Basal Ganglia Network Integration in Schizophrenia.* Front Hum Neurosci, 2015. **9**: p. 561.

44. Wang, D., et al., *Altered functional connectivity of the cingulate subregions in schizophrenia.* Transl Psychiatry, 2015. **5**(6): p. e575.

45. Xu, L., et al., *Selective Functional Disconnection of the Dorsal Subregion of the Temporal Pole in Schizophrenia.* Sci Rep, 2015. **5**: p. 11258.

46. Zhou, Y., et al., *The selective impairment of resting-state functional connectivity of the lateral subregion of the frontal pole in schizophrenia.* PLoS One, 2015. **10**(3): p. e0119176.

47. Chen, X., et al., *Functional abnormalities of the right posterior insula are related to the altered self-experience in schizophrenia.* Psychiatry Research - Neuroimaging, 2016. **256**: p. 26-32.

48. Liu, X., et al., *Selective functional connectivity abnormality of the transition zone of the inferior parietal lobule in schizophrenia.* Neuroimage Clin, 2016. **11**: p. 789-795.

49. Penner, J., et al., *Medial Prefrontal and Anterior Insular Connectivity in Early Schizophrenia and Major Depressive Disorder: A Resting Functional MRI Evaluation of Large-Scale Brain Network Models.* Front Hum Neurosci, 2016. **10**: p. 132.

50. Peters, H., et al., *Changes in extra-striatal functional connectivity in patients with schizophrenia in a psychotic episode.* British Journal of Psychiatry, 2017. **210**(1): p. 75-82.

51. Zhuo, C., et al., *Brain structural and functional dissociated patterns in schizophrenia.* BMC Psychiatry, 2017. **17**(1): p. 45.

52. Ferri, J., et al., *Resting-state thalamic dysconnectivity in schizophrenia and relationships with symptoms.* Psychol Med, 2018. **48**(15): p. 2492-2499.

53. Penner, J., et al., *Higher order thalamic nuclei resting network connectivity in early schizophrenia and major depressive disorder.* Psychiatry Res Neuroimaging, 2018. **272**: p. 7-16.

54. Penner, J., et al., *Temporoparietal Junction Functional Connectivity in Early Schizophrenia and Major Depressive Disorder.* Chronic Stress (Thousand Oaks), 2018. **2**: p. 2470547018815232.

55. Sharma, A., et al., *Altered resting state functional connectivity in early course schizophrenia.* Psychiatry Res Neuroimaging, 2018. **271**: p. 17-23.

56. Dong, D.B., et al., *Reconfiguration of Dynamic Functional Connectivity in Sensory and Perceptual System in Schizophrenia.* Cerebral Cortex, 2019. **29**(8): p. 3577-3589.

57. Yasuda, Y., et al., *Brain morphological and functional features in cognitive subgroups of schizophrenia.* Psychiatry Clin Neurosci, 2020. **74**(3): p. 191-203.
