# Supplementary material for: Meta-analysis of structural and functional brain abnormalities in schizophrenia with persistent negative symptoms using activation likelihood estimation
Source: Front Psychiatry. 2022 Sep 27;13:957685. doi: 10.3389/fpsyt.2022.957685 (PMC9552970; doi:10.3389/fpsyt.2022.957685)
Supplement: Supplementary file 2 [file Table_2.docx]

**Table S2. Results of the jackknife analysis in all included GMV studies.**

| **Study** | MFG.L | MFG.R | AMYG.L | IFG.L | ACG.L | STG.L | INS.L | INS.R | ACG.R |
| --- | --- | --- | --- | --- | --- | --- | --- | --- | --- |
| Paillère-Martinot^[1]^ | Yes | Yes | Yes | Yes | No | Yes | Yes | Yes | Yes |
| Sigmundsson^[2]^ | Yes | Yes | No | Yes | Yes | Yes | Yes | Yes | Yes |
| Kawasaki^[3]^ | Yes | Yes | Yes | No | Yes | Yes | Yes | Yes | Yes |
| Jayakumar^[4]^ | Yes | Yes | No | Yes | Yes | Yes | Yes | Yes | Yes |
| Bassitt^[5]^ | Yes | Yes | Yes | No | Yes | Yes | Yes | Yes | Yes |
| Koutsouleris^[6]^ | Yes | Yes | Yes | No | Yes | Yes | Yes | Yes | Yes |
| Meisenzahl^[7]^ | Yes | Yes | Yes | Yes | No | Yes | Yes | Yes | Yes |
| Herold^[8]^ | Yes | Yes | Yes | Yes | Yes | Yes | Yes | Yes | Yes |
| Whitford^[9]^ | Yes | Yes | Yes | Yes | Yes | Yes | Yes | Yes | Yes |
| Cascella^[10]^ | Yes | Yes | Yes | Yes | Yes | Yes | Yes | Yes | Yes |
| Anderson^[11]^ | Yes | Yes | No | Yes | Yes | Yes | Yes | Yes | Yes |
| Huang,P^[12]^ | Yes | Yes | Yes | Yes | Yes | Yes | Yes | Yes | Yes |
| Poletti^[13]^ | Yes | Yes | Yes | No | Yes | Yes | Yes | Yes | Yes |
| Huang,X^[14]^ | Yes | Yes | Yes | Yes | Yes | Yes | Yes | Yes | Yes |
| Kim,G. W^[15]^ | Yes | Yes | Yes | Yes | Yes | Yes | Yes | Yes | Yes |
| Kuroki^[16]^ | Yes | Yes | No | Yes | Yes | Yes | Yes | Yes | Yes |
| Szendi^[17]^ | Yes | Yes | Yes | Yes | Yes | Yes | Yes | Yes | Yes |
| Spalthoff^[18]^ | Yes | Yes | Yes | Yes | Yes | Yes | Yes | Yes | Yes |
| Zhao^[19]^ | Yes | Yes | Yes | Yes | Yes | Yes | Yes | Yes | Yes |
| Lei^[20]^ | Yes | Yes | Yes | Yes | Yes | Yes | Yes | Yes | Yes |
| Neugebauer^[21]^ | Yes | Yes | Yes | No | Yes | No | Yes | Yes | Yes |
| **Total** | 21/21 | 21/21 | 17/21 | 16/21 | 19/21 | 20/21 | 21/21 | 21/21 | 21/21 |

Abbreviations: GMV, gray matter volume; MFG, medial frontal gyrus; AMYG, amygdala; IFG, inferior frontal gyrus; STG, superior temporal gyrus; INS, insula; ACG, anterior cingulate gyrus; R, right; L, left.

**Table S3. Results of the jackknife analysis in all included FC studies.**

| **Study** | **Decreased FC** | | | |  | **Increased FC** | | | | | |
| --- | --- | --- | --- | --- | --- | --- | --- | --- | --- | --- | --- |
|  | MFG.L | SFG.L | CG.L | CG.R |  | PCUN.L | THA.L | PCG.R | PCUN.R | CUN.R | THA.R |
| Bluhm^[22]^ | Yes | Yes | Yes | Yes |  |  |  |  |  |  |  |
| Fan,F.M^[23]^ | Yes | Yes | Yes | Yes |  | Yes | Yes | Yes | Yes | Yes | Yes |
| Chang,X^[24]^ | Yes | Yes | Yes | Yes |  | No | Yes | Yes | Yes | Yes | Yes |
| Manoliu^[25]^ | Yes | Yes | Yes | Yes |  | No | Yes | No | No | No | Yes |
| Zhuo,C^[26]^ | Yes | Yes | Yes | Yes |  | Yes | Yes | Yes | Yes | Yes | Yes |
| Alonso-Solís^[27]^ | Yes | Yes | Yes | Yes |  | Yes | Yes | Yes | Yes | Yes | Yes |
| Chang,X^[28]^ | Yes | Yes | Yes | Yes |  |  |  |  |  |  |  |
| Duan,M^[29]^ | Yes | No | Yes | Yes |  | Yes | Yes | Yes | Yes | Yes | Yes |
| Wang,D^[30]^ | No | Yes | Yes | No |  | No | Yes | No | No | No | Yes |
| Xu,L^[31]^ | Yes | Yes | Yes | Yes |  |  |  |  |  |  |  |
| Zhou,Y^[32]^ | Yes | Yes | Yes | Yes |  |  |  |  |  |  |  |
| Chen,X^[33]^ | Yes | Yes | Yes | Yes |  | Yes | Yes | Yes | Yes | Yes | Yes |
| Liu, X^[34]^ |  |  |  |  |  | Yes | Yes | Yes | Yes | Yes | Yes |
| Penner,J^[35]^ | Yes | Yes | Yes | Yes |  |  |  |  |  |  |  |
| Peters^[36]^ | Yes | Yes | Yes | Yes |  |  |  |  |  |  |  |
| Zhuo,C^[37]^ | Yes | Yes | Yes | Yes |  | Yes | No | Yes | Yes | Yes | No |
| Ferri.^[38]^ |  |  |  |  |  | Yes | Yes | Yes | Yes | Yes | Yes |
| Penner,J.a^[39]^ | Yes | Yes | Yes | Yes |  | Yes | Yes | Yes | Yes | Yes | Yes |
| Penner,J.b^[40]^ | Yes | Yes | Yes | Yes |  |  |  |  |  |  |  |
| Sharma^[41]^ | Yes | Yes | Yes | Yes |  |  |  |  |  |  |  |
| Dong^[42]^ |  |  |  |  |  | Yes | No | Yes | Yes | Yes | No |
| Yasuda^[43]^ | Yes | Yes | Yes | Yes |  | No | No | No | No | No | Yes |
| **Total** | 18/19 | 18/19 | 19/19 | 18/19 |  | 10/14 | 11/14 | 11/14 | 11/14 | 11/14 | 12/14 |

Abbreviations: FC, functional connectivity; MFG, medial frontal gyrus; SFG, superior frontal gyrus; CG, cingulate gyrus; PCG, posterior cingulate gyrus; PCUN, precuneus; THA, thalamus; CUN, cuneus; R, right; L, left.

1. Paillère-Martinot, M., et al., *Cerebral gray and white matter reductions and clinical correlates in patients with early onset schizophrenia.* Schizophr Res, 2001. **50**(1-2): p. 19-26.

2. Sigmundsson, T., et al., *Structural abnormalities in frontal, temporal, and limbic regions and interconnecting white matter tracts in schizophrenic patients with prominent negative symptoms.* Am J Psychiatry, 2001. **158**(2): p. 234-43.

3. Kawasaki, Y., et al., *Structural brain differences in patients with schizophrenia and schizotypal disorder demonstrated by voxel-based morphometry.* Eur Arch Psychiatry Clin Neurosci, 2004. **254**(6): p. 406-14.

4. Jayakumar, P.N., et al., *Optimized voxel-based morphometry of gray matter volume in first-episode, antipsychotic-naive schizophrenia.* Prog Neuropsychopharmacol Biol Psychiatry, 2005. **29**(4): p. 587-91.

5. Bassitt, D.P., et al., *Insight and regional brain volumes in schizophrenia.* Eur Arch Psychiatry Clin Neurosci, 2007. **257**(1): p. 58-62.

6. Koutsouleris, N., et al., *Structural correlates of psychopathological symptom dimensions in schizophrenia: a voxel-based morphometric study.* Neuroimage, 2008. **39**(4): p. 1600-12.

7. Meisenzahl, E.M., et al., *Structural brain alterations at different stages of schizophrenia: A voxel-based morphometric study.* Schizophrenia Research, 2008. **104**(1-3): p. 44-60.

8. Herold, R., et al., *Regional gray matter reduction and theory of mind deficit in the early phase of schizophrenia: a voxel-based morphometric study.* Acta Psychiatr Scand, 2009. **119**(3): p. 199-208.

9. Whitford, T.J., et al., *Delusions and dorso-medial frontal cortex volume in first-episode schizophrenia: A voxel-based morphometry study.* Psychiatry Research-Neuroimaging, 2009. **172**(3): p. 175-179.

10. Cascella, N.G., et al., *Gray-matter abnormalities in deficit schizophrenia.* Schizophr Res, 2010. **120**(1-3): p. 63-70.

11. Anderson, V.M., et al., *Extensive Gray Matter Volume Reduction in Treatment-Resistant Schizophrenia.* International Journal of Neuropsychopharmacology, 2015. **18**(7).

12. Huang, P., et al., *Decreased bilateral thalamic gray matter volume in first-episode schizophrenia with prominent hallucinatory symptoms: A volumetric MRI study.* Sci Rep, 2015. **5**: p. 14505.

13. Poletti, S., et al., *Adverse childhood experiences influence the detrimental effect of bipolar disorder and schizophrenia on cortico-limbic grey matter volumes.* J Affect Disord, 2016. **189**: p. 290-7.

14. Huang, X., et al., *Decreased Left Putamen and Thalamus Volume Correlates with Delusions in First-Episode Schizophrenia Patients.* Front Psychiatry, 2017. **8**: p. 245.

15. Kim, G.W., Y.H. Kim, and G.W. Jeong, *Whole brain volume changes and its correlation with clinical symptom severity in patients with schizophrenia: A DARTEL-based VBM study.* PLoS One, 2017. **12**(5): p. e0177251.

16. Kuroki, N., et al., *Brain structure differences among male schizophrenic patients with history of serious violent acts: an MRI voxel-based morphometric study.* BMC Psychiatry, 2017. **17**(1): p. 105.

17. Szendi, I., et al., *A New Division of Schizophrenia Revealed Expanded Bilateral Brain Structural Abnormalities of the Association Cortices.* Front Psychiatry, 2017. **8**: p. 127.

18. Spalthoff, R., C. Gaser, and I. Nenadić, *Altered gyrification in schizophrenia and its relation to other morphometric markers.* Schizophr Res, 2018. **202**: p. 195-202.

19. Zhao, C.a., et al., *Structural and functional brain abnormalities in schizophrenia: A cross-sectional study at different stages of the disease.* Progress in Neuro Psychopharmacology & Biological Psychiatry, 2018. **83**: p. 27-32.

20. Lei, W., et al., *Progressive brain structural changes after the first year of treatment in first-episode treatment-naive patients with deficit or nondeficit schizophrenia.* Psychiatry Res Neuroimaging, 2019. **288**: p. 12-20.

21. Neugebauer, K., et al., *Nerve Growth Factor Serum Levels Are Associated With Regional Gray Matter Volume Differences in Schizophrenia Patients.* Front Psychiatry, 2019. **10**: p. 275.

22. Bluhm, R.L., et al., *Spontaneous low-frequency fluctuations in the BOLD signal in schizophrenic patients: anomalies in the default network.* Schizophr Bull, 2007. **33**(4): p. 1004-12.

23. Fan, F.M., et al., *Ventral medial prefrontal functional connectivity and emotion regulation in chronic schizophrenia: a pilot study.* Neurosci Bull, 2013. **29**(1): p. 59-74.

24. Chang, X., et al., *Altered default mode and fronto-parietal network subsystems in patients with schizophrenia and their unaffected siblings.* Brain Res, 2014. **1562**: p. 87-99.

25. Manoliu, A., et al., *Aberrant dependence of default mode/central executive network interactions on anterior insular salience network activity in schizophrenia.* Schizophr Bull, 2014. **40**(2): p. 428-37.

26. Zhuo, C., et al., *Functional connectivity density alterations in schizophrenia.* Front Behav Neurosci, 2014. **8**: p. 404.

27. Alonso-Solís, A., et al., *Resting-state functional connectivity alterations in the default network of schizophrenia patients with persistent auditory verbal hallucinations.* Schizophr Res, 2015. **161**(2-3): p. 261-8.

28. Chang, X., et al., *Distinct inter-hemispheric dysconnectivity in schizophrenia patients with and without auditory verbal hallucinations.* Sci Rep, 2015. **5**: p. 11218.

29. Duan, M., et al., *Altered Basal Ganglia Network Integration in Schizophrenia.* Front Hum Neurosci, 2015. **9**: p. 561.

30. Wang, D., et al., *Altered functional connectivity of the cingulate subregions in schizophrenia.* Transl Psychiatry, 2015. **5**(6): p. e575.

31. Xu, L., et al., *Selective Functional Disconnection of the Dorsal Subregion of the Temporal Pole in Schizophrenia.* Sci Rep, 2015. **5**: p. 11258.

32. Zhou, Y., et al., *The selective impairment of resting-state functional connectivity of the lateral subregion of the frontal pole in schizophrenia.* PLoS One, 2015. **10**(3): p. e0119176.

33. Chen, X., et al., *Functional abnormalities of the right posterior insula are related to the altered self-experience in schizophrenia.* Psychiatry Research - Neuroimaging, 2016. **256**: p. 26-32.

34. Liu, X., et al., *Selective functional connectivity abnormality of the transition zone of the inferior parietal lobule in schizophrenia.* Neuroimage Clin, 2016. **11**: p. 789-795.

35. Penner, J., et al., *Medial Prefrontal and Anterior Insular Connectivity in Early Schizophrenia and Major Depressive Disorder: A Resting Functional MRI Evaluation of Large-Scale Brain Network Models.* Front Hum Neurosci, 2016. **10**: p. 132.

36. Peters, H., et al., *Changes in extra-striatal functional connectivity in patients with schizophrenia in a psychotic episode.* British Journal of Psychiatry, 2017. **210**(1): p. 75-82.

37. Zhuo, C., et al., *Brain structural and functional dissociated patterns in schizophrenia.* BMC Psychiatry, 2017. **17**(1): p. 45.

38. Ferri, J., et al., *Resting-state thalamic dysconnectivity in schizophrenia and relationships with symptoms.* Psychol Med, 2018. **48**(15): p. 2492-2499.

39. Penner, J., et al., *Higher order thalamic nuclei resting network connectivity in early schizophrenia and major depressive disorder.* Psychiatry Res Neuroimaging, 2018. **272**: p. 7-16.

40. Penner, J., et al., *Temporoparietal Junction Functional Connectivity in Early Schizophrenia and Major Depressive Disorder.* Chronic Stress (Thousand Oaks), 2018. **2**: p. 2470547018815232.

41. Sharma, A., et al., *Altered resting state functional connectivity in early course schizophrenia.* Psychiatry Res Neuroimaging, 2018. **271**: p. 17-23.

42. Dong, D.B., et al., *Reconfiguration of Dynamic Functional Connectivity in Sensory and Perceptual System in Schizophrenia.* Cerebral Cortex, 2019. **29**(8): p. 3577-3589.

43. Yasuda, Y., et al., *Brain morphological and functional features in cognitive subgroups of schizophrenia.* Psychiatry Clin Neurosci, 2020. **74**(3): p. 191-203.
